# BaFe_1−x_Cu_x_O_3_ Perovskites as Active Phase for Diesel (DPF) and Gasoline Particle Filters (GPF)

**DOI:** 10.3390/nano9111551

**Published:** 2019-10-31

**Authors:** Verónica Torregrosa-Rivero, Carla Moreno-Marcos, Vicente Albaladejo-Fuentes, María-Salvadora Sánchez-Adsuar, María-José Illán-Gómez

**Affiliations:** Carbon Materials and Environment Research Group, Department of Inorganic Chemistry, Faculty of Science, University of Alicante, Av. Alicante s/n, San Vicente del Raspeig, 03690 Alicante, Spain; vero.torregrosa@ua.es (V.T.-R.); carlamorenomarcos1@gmail.com (C.M.-M.); vicentealbaladejo@gmail.com (V.A.-F.); dori@ua.es (M.-S.S.-A.)

**Keywords:** Iron-based perovskites, copper, NO oxidation to NO_2_, NO_2_-assisted diesel soot oxidation, soot oxidation under GDI exhaust conditions

## Abstract

BaFe_1−x_Cu_x_O_3_ perovskites (*x* = 0, 0.1, 0.3 and 0.4) have been synthetized, characterized and tested for soot oxidation in both Diesel and Gasoline Direct Injection (GDI) exhaust conditions. The catalysts have been characterized by BET, ICP-OES, SEM-EDX, XRD, XPS, H_2_-TPR and O_2_-TPD and the results indicate the incorporation of copper in the perovskite lattice which leads to: (i) the deformation of the initial hexagonal perovskite structure for the catalyst with the lowest copper content (BFC1), (ii) the modification to cubic from hexagonal structure for the high copper content catalysts (BFC3 and BFC4), (iii) the creation of a minority segregated phase, BaO_x_-CuO_x_, in the highest copper content catalyst (BFC4), (iv) the rise in the quantity of oxygen vacancies/defects for the catalysts BFC3 and BFC4, and (v) the reduction in the amount of O_2_ released in the course of the O_2_-TPD tests as the copper content increases. The BaFe_1−x_Cu_x_O_3_ perovskites catalyze both the NO_2_-assisted diesel soot oxidation (500 ppm NO, 5% O_2_) and, to a lesser extent, the soot oxidation under fuel cuts GDI operation conditions (1% O_2_). BFC0 is the most active catalysts as the activity seems to be mainly related with the amount of O_2_ evolved during an. O_2_-TPD, which decreases with copper content.

## 1. Introduction

The high toxicity of particulate matter (PM) or soot, mainly produced by internal combustion engines, is well established. As in Europe the transport sector generates a 14% of PM2.5 (particulates with a size lesser than 2.5 m, the most hazardous portion), the actual European emissions legislation (Euro 6c) for new passengers vehicles meet or decreases the Particulates Numbers (PN) generated by Gasoline Direct Injection (GDI) to the level corresponding to Diesel engines [1]. GDI engines are considered more effective than diesel engines due to the substantial decrease of fuel intake and CO_2_ emissions [2]. Consequently, a growth in the US and European market of GDI cars is being observed. To attend the actual European emission legislation, the use of Gasoline Particulate Filter (GPF) is necessary for GDI vehicles, as the Diesel Particulate Filter (DPF) was for Diesel vehicles. In both filters, periodic regeneration is demanded to avoid soot accumulation in the channels of the filter [3,4,5]. 

In Diesel engine, as NO_2_ promotes soot oxidation, a catalyst able to oxidize NO to NO_2_ is incorporated into the DPF to carry out the NO_2_-assisted soot oxidation. In fact, several systems (most of them containing Platinum Group Metals, PGM) were developed and implemented in diesel cars to oxidize soot. However, recently, the EU [6] has highlighted that the use of critical raw materials (such as PGM) must be optimized.

Based on the success of DPF in diesel engines, GPF is proposed as a solution for GDI engines. The operating requirements of GPF differ largely from those of the DPF, as NO_2_ is not present and a very low amount of O_2_ is available in the GDI exhaust downstream the TWC [7,8,9]. Thus, active catalysts to oxidize soot in poor (or even null) oxygen conditions must be developed. However, even though it is a challenging issue, the soot oxidation reaction in the severe GDI exhaust requirements (i.e., <10,000 ppm of O_2_) has been scarcely studied [8,9,10]. 

Among the catalysts suggested for O_2_-soot oxidation [8,9,10,11,12,13,14,15,16,17,18], one of the most interesting are mixed oxides with perovskite structure (ABO_3_), as their properties can be tailored by selecting the nature of the A and B ions according to the specific needs of the oxidation reaction [19]. Certainly, perovskites are an option with future potential as soot oxidation catalysts in DPF conditions [16,17,18,19,20,21,22,23,24,25,26], as well as for other oxidation reactions such as CO, hydrocarbons and volatile organic compounds [27,28,29,30,31,32,33]. In previous papers [26,34], the beneficial result of the incorporation of copper into the structure of BaMnO_3_ and BaTiO_3_ perovskites for NO_2_-assisted diesel soot oxidation was explored. Lately, Hernández et al. [8,9] stated that iron-based perovskites are also appealing as soot oxidation catalysts in GDI exhaust requirements.

Considering this background, and taking into account the promising performance previously featured by a BaFe_1−x_Cu_x_O_3_ catalysts series for soot oxidation in the most severe GDI exhaust requirements (regular stoichiometric GDI operation, i.e., 0% O_2_) [35], the objective of this research is to further study the influence of the partial replacement of iron by copper in the properties of a BaFeO_3_ perovskite which will define its catalytic performance for soot oxidation. Therefore, BaFe_1−x_Cu_x_O_3_ catalysts (*x* = 0, 0.1, 0.3 and 0.4) were synthetized, characterized and tested for soot oxidation in both diesel and “fuel cuts” GDI exhaust conditions (i.e., 1% O_2_). 

## 2. Materials and Methods 

### 2.1. Catalyst Preparation

BaFe_1−x_Cu_x_O_3_ catalysts (*x* = 0, 0.1, 0.3, 0.4) have been obtained using a citrate sol-gel method [26]. Ba(CH_3_COOH)_2_ (Sigma-Aldrich, 99%), Fe(NO_3_)_2_·9H_2_O (Sigma-Aldrich, 97%) and Cu(NO_3_)_2_·3H_2_O (Panreac, 99%) have been employed as metal precursors. Briefly, a 1M citric acid solution, with a 1:2 molar ratio with respect to barium has been heated to 60 °C. The solution pH has been raised to 8.5 with ammonia solution. Subsequently, the corresponding amounts of barium, iron, and copper precursors have been incorporated, and the pH value has been readjusted to 8.5 with ammonia solution. The solution was hold at 65 °C during 5 h and later dried at 90 °C for 48 h. The dried gel has been calcined at 150 °C for 1 h and then, at 850 °C 6 h [26]. Table 1 includes the catalysts nomenclature.

### 2.2. Characterization

To measure the metal content in the samples by Inductively Coupled Plasma Atomic Emission Spectroscopy (ICP-OES), a Perkin-Elmer equipment (Optima 4300 DV) has been used. For the analysis, copper was extracted dissolving the samples with magnetic stirring in 8M HCl solution by reflux heating. 

An Autosorb-6B instrument (Quantachrome Instruments, Boynton Beach, FL, USA) was used to determine, by N_2_ adsorption at −196 °C, the Brunauer Emmet Teller (BET) surface area of the samples, which were previously degasified at 250 °C for 4 h.

X-ray diffraction (XRD) tests were performed between 20–80° 2θ angles with a step rate of 1.5°/2 min and using CuKα (0.15418 nm) radiation in a Bruker D8-Advance device. The Rietveld analysis of XRD data was developed with the Automatic Rietveld Refinement (HIGHScore Plus from PANalytical program).

A ZEISS Merlin VP Compact Field Emission Scanning Electron Microscopy (FESEM) equipment (Quantax 400 from Bruker, Berlin, Germany) was employed to analyze the morphology of the catalysts and to determine the elemental composition of the catalysts (by Energy Dispersive X-Ray analysis, EDX). 

X-Ray Photoelectron Spectroscopy (XPS) was used to obtain the surface composition. To register the XPS spectra, a K-Alpha photoelectron spectrometer by Thermo-Scientific, with an Al Kα (1486.6 eV) radiation source, was used in the following conditions: 5 × 10^−10^ mbar pressure in the chamber and setting the C1s transition at 284.6 eV. The binding energy (BE) and kinetic energy (KE) values were then determined with the peak-fit software of the spectrophotometer, to regulate the BE and KE scales.

Reducibility of the catalysts was evaluated by Temperature Programmed Reduction with H_2_ (H_2_-TPR). The experiments were developed in a Pulse Chemisorb 2705 device from Micromeritics fitted with a Thermal Conductivity Detector (TCD to find out the outlet gas composition changes. 20 mg of the sample was heated at 10 °C/min from room temperature to 1000 °C in 5% H_2_/Ar atmosphere (40 mL/min, *P_t_* = 1 atm). The H_2_ consumption amount was determined using a CuO sample supplied by Micromeritics. 

### 2.3. Activity Tests

The catalytic activity for NO to NO_2_ oxidation and NO_2_-assisted diesel soot oxidation was established by Temperature Programmed Reaction (NOx-TPR) using of a gas mixture composed of 500 ppm NOx and 5% O_2_, balanced with N_2_ (500 mL/min gas flow). For NO oxidation experiments, 80 mg of the catalyst were mixed with SiC, in a 1:4 mass ratio, and warmed from 25 to 800 °C, at 10 °C/min, in a quartz fixed-bed reactor. The activity for diesel soot oxidation was evaluated adding 20 mg of Printex U from Degussa (employed as surrogated soot, which represents the least reactive fraction of particulate matter [8,9,10,16,26,34,35,36,37,38]), in loose contact with the catalyst. For the catalyst with the highest activity, isothermal soot oxidation reactions at 450 °C were also performed. The gas composition was monitored by specific Non-dispersive Infrared Ultraviolet NDIR-UV gas analyzers for NO, NO_2_, CO, CO_2_, and O_2_ (Rosemount Analytical Model BINOS 1001, 1004 and 100). The NO_2_ generation, soot conversion and CO_2_ selectivity percentages were calculated using Equations (1), (2), and (3), respectively:

(1) $${\mathrm{NO}}_{2}\left(\%\right)=\frac{{\mathrm{NO}}_{2,\;\mathrm{out}}}{{\mathrm{NO}}_{x,\mathrm{out}}}\;\times \;100$$

(2) $$\mathrm{Soot}\;\mathrm{conversion}\left(\%\right)=\frac{\sum_{0}^{t}{\mathrm{CO}}_{2}+\mathrm{CO}}{{\left({\mathrm{CO}}_{2}+\mathrm{CO}\right)}_{\mathrm{total}}}\;\times 100$$

(3) $${\mathrm{CO}}_{2}\;\mathrm{selectivity}\;\left(\%\right)=\frac{{\mathrm{CO}}_{2,\;\mathrm{total}}}{{\left({\mathrm{CO}}_{2}+\mathrm{CO}\right)}_{\mathrm{total}}}\;\times 100$$

where NO_2,out_ and NO_x_,_out_ are the NO_2_ and NOx concentrations determined at the reactor exit, $\overset{t}{\underset{0}{\sum }}{\mathrm{CO}}_{2}+\mathrm{CO}$ is the quantity of CO_2_ and CO evolved at a time *t*, and and ${\left({\mathrm{CO}}_{2}+\mathrm{CO}\right)}_{\mathrm{total}}$ are the CO and CO_2_ + CO evolved during all the experiment time. 

To determine the catalysts performance for soot oxidation in GDI exhaust conditions, a gas mixture with 1% O_2_ in He was used as it is the typical O_2_ concentration at the turbine-GDI engine exit, i.e., upstream the TWC [8], but also because it simulates the fuel cut GDI operation conditions (<20% O_2_) [7]. These experiments were developed as Temperature Programmed Reactions (6 °C/min from 150 °C temperature till 900 °C, 500 mL/min gas flow) in a quartz fixed-bed reactor using 80 mg of the catalysts and 20 mg of Printex-U (1:4 soot/catalyst ratio in loose contact) mixed with SiC. A Gas Chromatograph (Hewlet Packard 8690) with two packed columns (Porapak Q and Molecular Sieve 5a) connected to a Thermal Conductivity Detector (TCD) was used for the measure of the gas composition. Previous to the soot oxidation reaction, the catalysts were preheated in the reaction mixture (1% O_2_ in He) at 150 °C during 1 hour. The soot conversion and CO_2_ selectivity percentages were calculated using Equations (2) and (3), respectively.

## 3. Results and Discussion

### 3.1. Characterization of the Fresh Catalysts

#### 3.1.1. Chemical, Morphological, and Structural Properties

Table 1 features the real copper content and BET surface area of the BaFe_1−x_Cu_x_O_3_ (*x* = 0, 0.1, 0.3, 0.4) perovskites obtained by ICP-OES and N_2_ adsorption, respectively. All the BaFe_1−x_Cu_x_O_3_ catalysts present a very low surface area, as it corresponds to mixed oxides with perovskite structure [19]. The data of the real copper content (very close to the nominal corresponding to the stoichiometric formula) reveal that nearly all the copper used in the synthesis appears in the catalysts. Concerning morphology, FESEM images (Figure S1 in Supplementary Information) show that catalysts are formed by highly agglomerated irregular grains with a size in the range of micrometer. The presence of a low amount of copper (BFC1 and BFC3) does not significantly change the morphology of the bare perovskite; however, for the catalyst with highest copper content (BFC4), a different type of grains is detected which could correspond to a new phase. The EDX data (see Table S1 in Supplementary Information) reveal an identical atomic percentage of Ba and Fe for BFC0, as expected according to perovskite composition (BaFeO_3_). However, for BFC4, in addition to the presence of Cu, larger atomic percentages of Ba and O are detected. This fact supports the existence of a new phase composed by barium, oxygen, and copper in the surface of this catalyst. 

**Figure 1:**
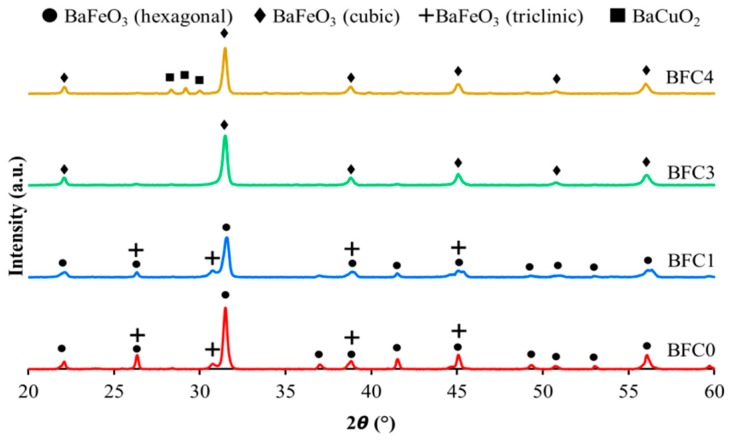
XRD patterns for fresh BaFe_1−x_Cu_x_O_3_ catalysts.

Figure 1 features the XRD profiles, showing (as expected according to the calculated t values shown in Table 1) a perovskite structure as an almost unique crystalline phase for all catalysts. Additionally, a Fe(III) and Fe(IV) mixed-oxide with triclinic structure is identified as a minority phase for BFC0 and BFC1, while, for BFC4, a BaO_x_-CuO_x_ phase (with a suggested stoichiometry of BaCuO_2_) appears. This oxide could correspond to the different type of grains observed by FESEM for BFC4 catalyst (Figure S1d in Supplementary Information) and justifies the EDX data (Table S1 in Supplementary Information) for this catalyst.

**Figure 2:**
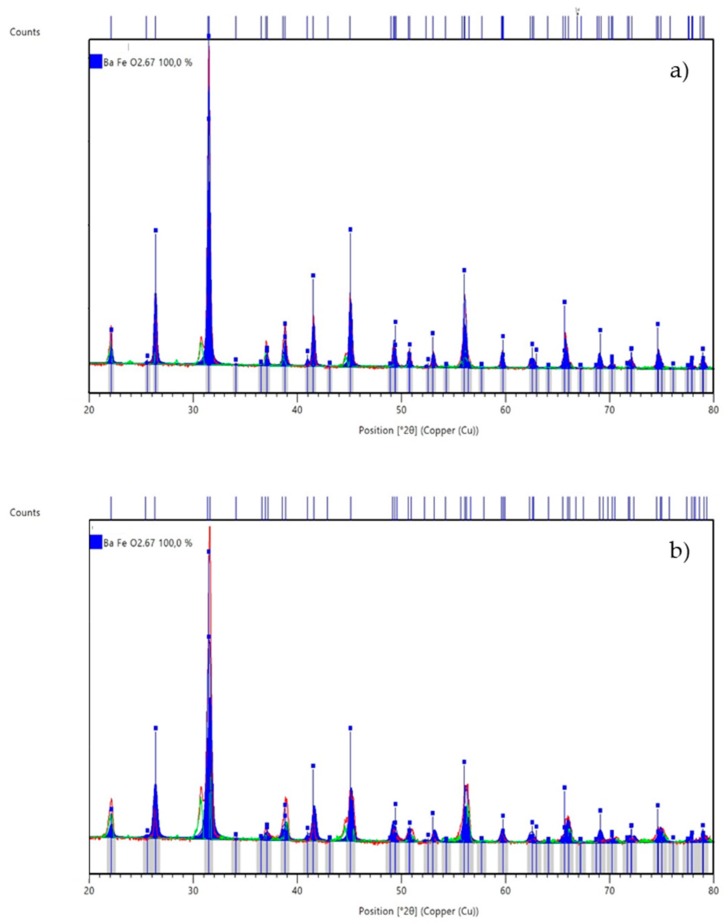

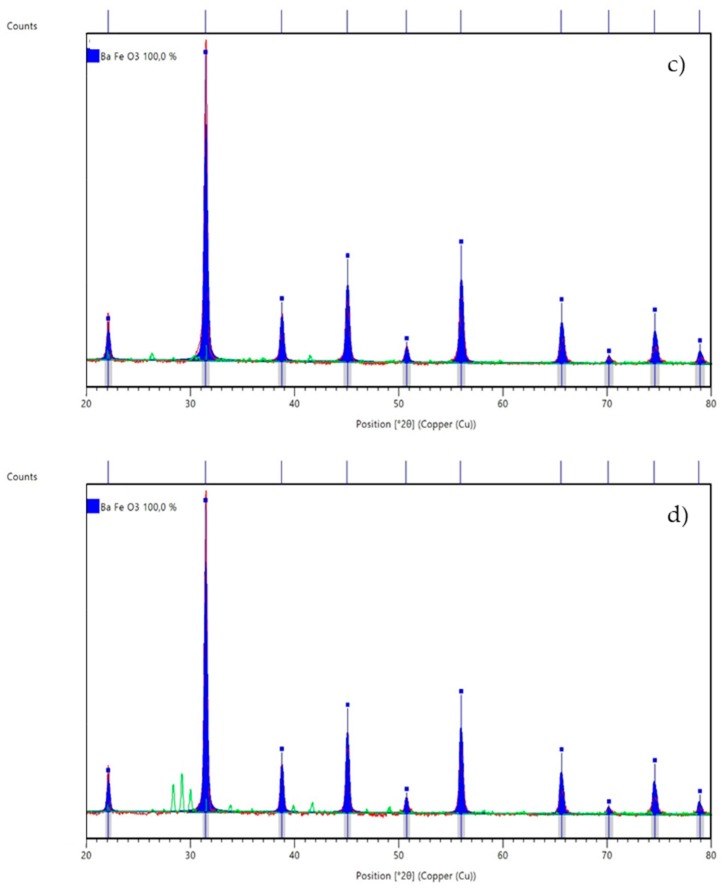
Rietveld analysis for (minority phase not included in the analysis): (**a**) BFC0: in red the original XRD pattern, in blue the Rietveld simulation corresponding to hexagonal perovskite structure and in green the residual data corresponding to triclinic structure; (**b**) BFC1: in red the original XRD pattern, in blue the Rietveld simulation corresponding to hexagonal perovskite structure and in green the residual data corresponding to triclinic structure; (**c**) BFC3: in red the original XRD pattern, in blue the Rietveld simulation corresponding to cubic perovskite structure and in green the residual data corresponding to BaCuO_2_; (**d**) BFC4: in red the original XRD pattern, in blue the Rietveld simulation corresponding to cubic perovskite structure and in green the residual data corresponding to BaCuO_2_.

For BFC0 and BFC1 catalysts, the diffraction peaks are assigned to a hexagonal perovskite structure; however, for BFC3 and BFC4, the peaks are consistent with a cubic structure. These results agree with the decrease in the *t* parameter values (Table 1), which becoming closer to 1 (corresponding to an ideal cubic structure) as the copper content increases. This structural modification (which has been verified by the Rietveld analysis presented in Figure 2 was previously noticed for other barium-based perovskites [26,34,38] and also for Sn- doped BaFeO_3_ perovskites [39], and supports that Cu has been introduced into the perovskite lattice. Concerning BFC1, the reduction in the intensity of the main perovskite peak (at. 31.5°) evidences that copper has been inserted into the perovskite structure [26,34,38]. Moreover, except for the catalyst with a highest copper content (BFC4), peaks corresponding to a copper segregated phase are not clearly identified, revealing that copper species are not segregated or, if they are, they would present a size under the detection limit of XRD. Finally, for BFC4, the presence of the BaO_x_-CuO_x_ phase as minority segregated phase shows a limit in the amount of copper introduced into the perovskite framework [26,34,35,38]. 

The average crystal size for the catalyst has been determined from the Full Width at Half Maximum (FWHM) of the main perovskite XRD peak (in hexagonal or cubic structure) applying the Scherrer equation [40]; data are included in Table 2. The average crystal size is smaller for the catalyst containing copper with hexagonal structure (BFC1) than for the bare perovskite (BFC0). On the contrary, for catalysts with cubic structure (BFC3 and BFC4), the average crystal size increases with the copper content. The lattice parameter for hexagonal (*a* and *c*) and cubic (*a*) perovskites have also been estimated from XRD data (Table 2). As the average crystal size, the decrease in *a* and *c* values is observed in the presence of copper for the catalyst with hexagonal structure (BFC1), confirming that copper has been inserted into the lattice. However, as the ionic radii of copper (as Cu^2+^, 0.73 **Å**) is larger than the Fe^3+^ ionic radii (0.65 **Å**) or Fe^4+^ (0.59 **Å**), an increase in the lattice parameters would be expected if this was the unique factor affecting the values. Nevertheless, it has been reported that a modification in the amount of the oxygen vacancies affects the lattice parameter [41], thus, it seems that the amount of oxygen vacancies is also being affected by copper incorporation into the BaFeO_3_ perovskite framework. For cubic perovskites (BFC3 and BFC4), the lattice parameter is almost constant but larger than the corresponding to a reference BaFeO_3_ with cubic structure (4.012 **Å**) [39], again supporting that copper has been inserted into the lattice. 

Summarizing, from XRD data, it can be concluded that copper is inserted into the perovskite structure causing: (i) the distortion of the original hexagonal perovskite structure for the catalyst with the lowest copper content (BFC1), (ii) the modification from hexagonal to cubic structure for the catalysts with high copper content (BFC3 and BFC4), (iii) the formation of a BaOx-CuOx oxide as minority segregated phase for BFC4 catalyst, and iv) a possible increase in the amount of oxygen vacancies/defects.

#### 3.1.2. Surface Properties

**Figure 3:**
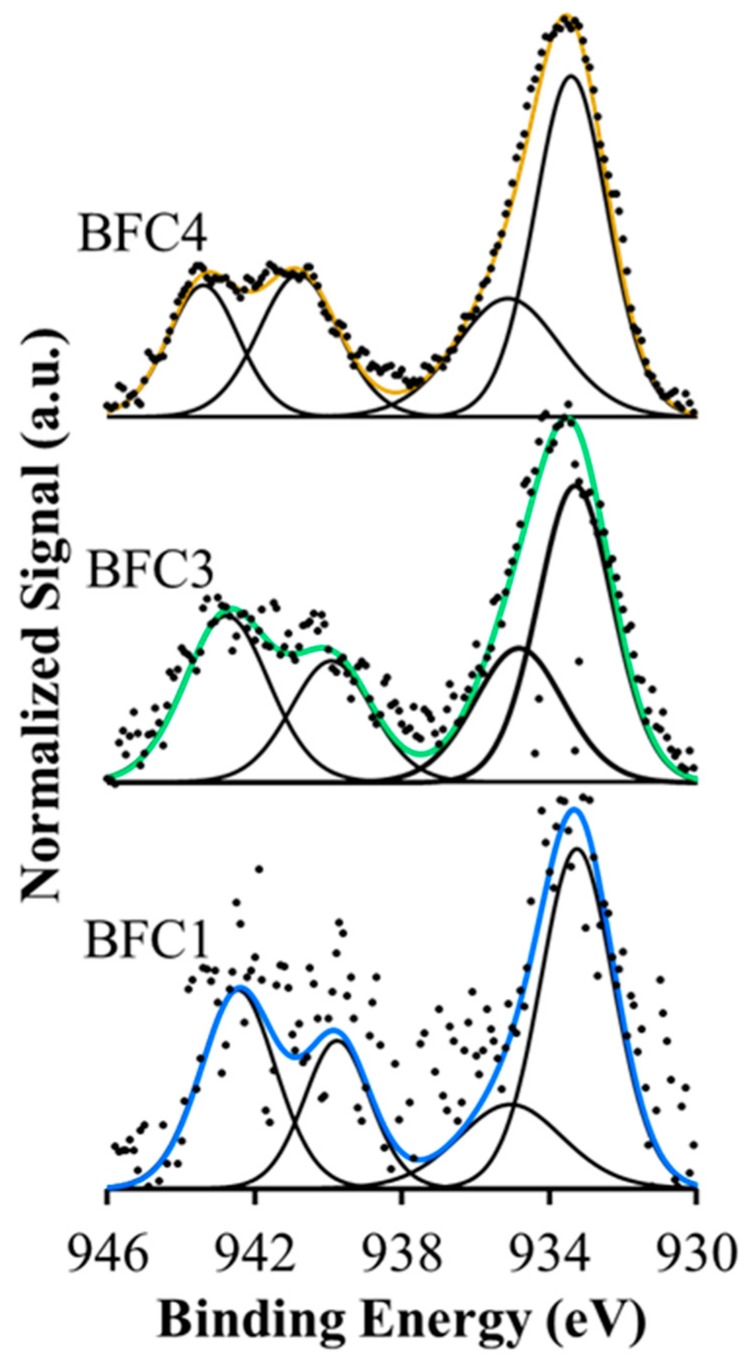
XPS spectra for Cu2p^3/2^ transition.

XPS analysis provides data about the surface composition of the BaFe_1−x_Cu_x_O_3_ perovskite catalysts. The XPS profiles corresponding to the Cu 2p^3/2^ transition are presented in Figure 3. Reduced copper species, such as metallic copper or Cu_2_O, usually appear at a binding energy (BE) close to 933 eV, while, for Cu(II) species, the Cu 2p^3/2^ transition appears above 933 eV [36,42,43,44]. In Figure 3, the BE maximum of the main XPS band appears slightly above 933 eV in the three catalysts containing copper, suggesting the presence of Cu(II) species. Moreover, Cu(I) and Cu(II) species can be distinguished by the presence of a satellite peak at 942–945 eV, due to an electron transfer from Cu 2p^3/2^ to 3d free level in Cu(II) [45]. The existence of the satellite peak for the three copper-content catalysts, that reveals the presence of Cu(II) species [45], confirms that copper is present as Cu(II) species. Additionally, based on the use of Auger data (Cu LMM) [45], the existence of Cu(II) species has been verified as reveals the Wagner (chemical state) plot shown in Figure S2 (Supplementary information). The deconvolution of the normalized Cu 2p^3/2^ bands reveals two contributions with maxima at around 933 eV and 935 eV, which seem to correspond to two different Cu(II) species [42,43,44]: (i) the band at lower BE, assignable to copper species with a weak electronic interaction with perovskite, that is, to CuO species located on the surface (Cu_S_) and (ii) the band at higher BE, corresponding to copper species with a strong electronic interaction with perovskite (Cu_L_), i.e., copper inserted in the lattice, near the surface. As the percentage of the area for the XPS band at 935 eV (Cu_L_ band) increases with the copper content from 26% to 33%, it seems that the presence of copper with a strong electronic interaction with perovskite is favored as copper content increases. However, a slight decrease of this value is observed for the BFC4 catalyst with respect to BFC3 (33% versus 35%), confirming that a limit for the copper insertion has been achieved. In fact, a comparison between the Cu/Cu+Fe+Ba ratio calculated by XPS and the corresponding nominal ratio (both data included in Table 3) confirms that copper has been inserted into the perovskite structure, as the XPS ratio are lower (for BFC1 and BFC3) or similar than (for BCF4) the nominal ratio [25,34,35,36,37,38]. It is remarkable that the smallest difference between these two values is presented by BFC4 catalyst, supporting, again, the limit in the copper insertion. Therefore, the copper which is not introduced into the lattice has to be dispersed on the surface forming the BaOx-CuOx phase, which was detected by XRD and EDX, as copper content is higher for BFC4 (Table 1).

**Figure 4:**
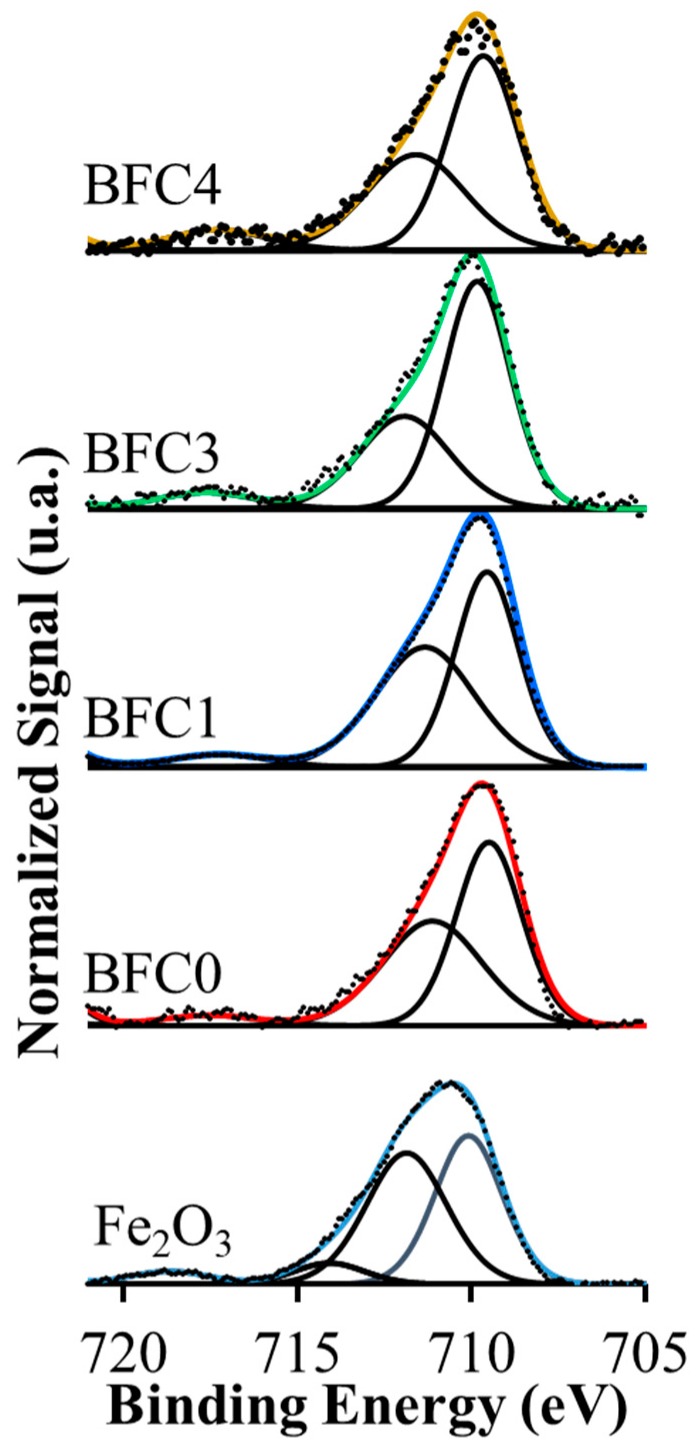
XPS spectra for Fe2p^3/2^ transition.

Figure 4 features the XPS spectra of the Fe 2p_3/2_ for BaFe_1−x_Cu_x_O_3_ catalysts and the corresponding to a Fe_2_O_3_ commercial sample use as reference. The maximum of the main XPS band for the four catalysts does not appear at exactly the same (BE) value than the corresponding to the reference suggesting the presence of Fe species with a different oxidation state or with the same oxidation state but in different proportion. The deconvolution of the main band shows two significant contributions at around 709 eV and 711 eV. Even though the identification of iron oxidation states by XPS is very difficult [46], according to literature [39,46,47,48,49,50], the first peak corresponds to Fe(III) species, and the second one could be assigned to Fe(IV) species [48,49,50]. It has been established that the position of the satellite peak is the key finger to detect the oxidation state of Fe [46,48]. Thus, the shake-up peak observed at 717 eV (which corresponds to the satellite peak of Fe(III)) supports the existence of this oxidation state [39,46,48,51]. However, the presence of Fe(IV) seems not to be supported by the XPS data, as the high BE peak at approximately 711 eV is not always unequivocally assigned to this oxidation state [46,48]. Thus, more evidence from other characterization techniques is needed to assume that Fe(IV) exits. The TPR-H_2_ results (see below) indicate that Fe(IV) and Fe(III) oxidation states co-exist in the BaFe_1−x_Cu_x_O_3_ catalysts, as it is observed that the experimental H_2_ consumption is in between the nominal (calculated) H_2_ consumption expected, considering that iron as Fe(III) or Fe(IV) is reduced to Fe(II). The presence of Fe(IV) in BaFe_1−x_Cu_x_O_3_ catalysts is additionally supported by the well-known stabilization of high oxidation state for B cation, as Fe(IV), in perovskites [19,39,48,49,50]. On the basis of the BaFeO_3_ stoichiometric formula, Fe(IV) must be the oxidation state for Fe in the perovskite, and, in the presence of copper, a rise in the Fe(IV) amount and /or the generation of additional oxygen vacancies into the perovskite structure would be expected to compensate the deficiency of positive charge due to the partial iron substitution [19]. In fact, the decrease in the lattice parameter observed by XRD for BFC1 with respect to BFC0 (Table 2) suggests an increase in the Fe(IV), which presents a lower ionic ratio that Fe(III). However, for BFC2 and BFC3, the lattice parameter (Table 2) increases revealing that the amount of Fe(IV) cannot be higher; thus, the generation of additional oxygen vacancies should be observed to balance the positive charge deficiency due to the increase of the copper content in the catalyst. This larger amount of oxygen vacancies has to cause the lattice expansion detected [41].

**Figure 5:**
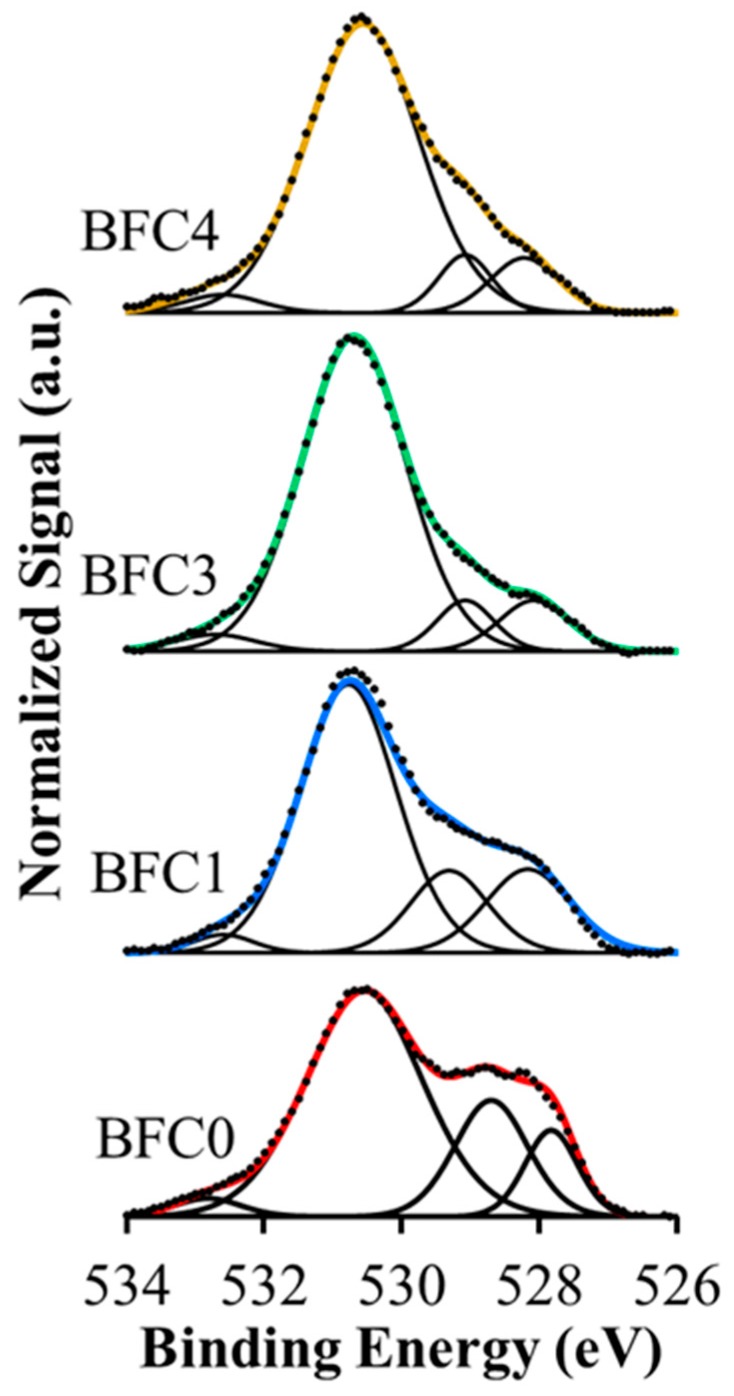
XPS spectra for O 1s transition.

Figure 5 presents the XPS spectra of the O1s transition for all catalysts, where three contributions are usually observed [36,42,43,44]: (i) at low BE (around 528 eV), corresponding to lattice oxygen (O_L_) in metal oxides, (ii) at intermediate BE (between 529 and 531 eV), assigned to adsorbed oxygen species such as, O_2_^−2^, surface CO_3_^−2^, and/or OH^−^ groups, and (iii) at high BE (533 eV approximately) due to oxygen in adsorbed water [52,53,54,55]. The intensity of the bands is modified in the presence of copper revealing changes in the amount of oxygen species on the catalysts surface. The values of O_L_/Cu+Ti+Ba ratio in Table 3 (determined from the peak area of O_L_, Fe2p^3/2^, Ba3d^3/2^, and Cu2p^3/2^ transitions) is higher for BFC1 than for the bare BFC0 perovskite, which means a lower amount of surface oxygen vacancies. This fact supports that the oxidation of Fe(III) to Fe(IV) occurs in the BFC1 perovskite to compensate the positive charge deficiency due to copper incorporation. However, for BFC3 and BFC4 catalysts, the lower O_L_/Cu+Ti+Ba ratio with respect to the nominal value confirms the generation of additional oxygen vacancies to balance the positive charge deficiency due to partial iron substitution by copper. Additionally, these results justify the change in the values of lattice parameters observed, that is: (i) the lower lattice parameters values for BFC1 catalyst with respect to BFC0 (Table 2) are due to the decrease in the amount of oxygen vacancies (Table 3), as, for this catalysts, the oxidation of Fe(III) to Fe(IV) takes place and (ii) the larger values for BFC3 and BFC4 (Table 2) are due to the rise in the amount of oxygen vacancies with respect to BFC0 (Table 3).

#### 3.1.3. Redox Properties

**Figure 6:**
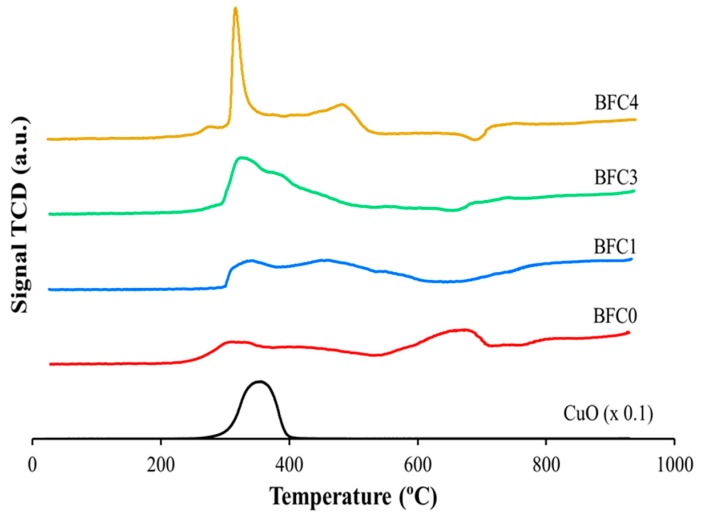
H_2_-TPR profiles for BaFe_1−x_Cu_x_O_3_ catalysts.

**Figure 7:**
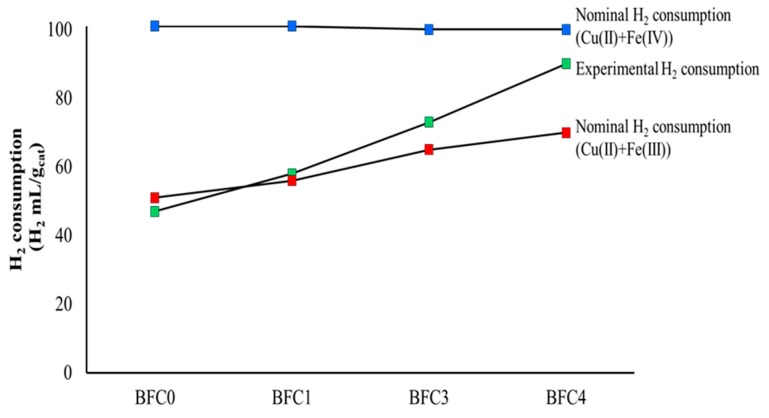
H_2_ consumption (mL/g of catalyst).

Reducibility and redox properties of the fresh BaFe_1−x_Cu_x_O_3_ catalysts were analyzed by Temperature Programmed Reduction with H_2_ (H_2_-TPR), which are the H_2_ consumption profiles shown in Figure 6. In Figure 7, the nominal (calculated) H_2_ consumption (mL of H_2_ per gram of catalysts) expected considering that iron, as Fe(III) or Fe(IV) in the perovskite, is reduced to Fe(II), is compared with the experimental H_2_ consumption determined from the H_2_-TPR profiles (Figure 6). It is observed that the experimental H_2_ consumption is in between both nominal values revealing that Fe(IV) and Fe(III) oxidation states co-exist in the BaFe_1−x_Cu_x_O_3_ catalysts. Furthermore, the experimental H_2_ consumption data indicates that the amount of Fe(IV) increases in the presence of copper.

In the complex H_2_ consumption profiles shown in Figure 6, three regions can be established [56,57,58]:

a) At low temperature, between approximately 200 °C and 550 °C, a broad H_2_ consumption signal is observed for all the catalyst that, according to literature, can be ascribed to different reduction processes: (i) the Cu(II) [34,38] reduction, (ii) the Fe(IV) and Fe(III) reduction to Fe(III) and Fe(II), as was observed for Fe_3_O_4_, and iii) the reduction of weakly chemisorbed oxygen upon surface oxygen vacancies of perovskite (α-oxygen) [34].
b) From around 550 °C to 700 °C, the H_2_ consumption peaks correspond to both the reduction of Fe(III) to Fe(II) as detected for the reduction of Fe_3_O_4_ to FeO and to the decomposition of surface oxygen species formed on oxygen vacancies (called α’-oxygen) [34], more strongly bonded to the perovskite than α-oxygen.
c) At high temperatures (T > 700 °C), broad TCD signals assigned to the reduction of Fe(II) to Fe(0) (causing the consequent destruction of the perovskite structure) could be found [56,57,58]. Nevertheless, the XRD data for catalysts after H_2_-TPR (not shown) reveal that the perovskite structure is still present, thus, the reduction to Fe(0) is not taking place and, consequently, H_2_ consumption is hardly observed at T > 700 °C. Therefore, the most relevant information related to the redox properties of the BaFe_1−x_Cu_x_O_3_ catalysts is located at T < 700 °C.

BFC0 profile shows two peaks at temperature lower than 700 °C: a broad peak with maximum at ca 300 °C and a more defined peak with a maximum at around 670 °C. The H_2_ consumption detected at temperature lower than 300 °C is usually related to the presence of Fe(IV) [56,57,58], supporting the existence of this oxidation state. The second H_2_ consumption peak, with a maximum at 670 °C, corresponds to the reduction of Fe(III) to Fe(II) and to desorption/reduction of oxygen surface species formed on oxygen vacancies (α’-oxygen) [34]. 

In the H_2_ consumption profile of BFC1 catalyst, a broad peak with two maxima, at approximately 350 °C and 450 °C, is identified. The first maximum is ascribed to the Cu(II) to Cu(0) reduction (appearing at lower temperature than the CuO used as a reference [38]) and also to the consumption due to the partial Fe(IV) and Fe(III) reduction to Fe(III) and Fe(II), respectively. The second maximum of this broad peak at 450 °C seems to correspond to: i) the reduction of Fe(III) to Fe(II), taking place at lower temperature than for the BaFeO_3_ catalyst, due to the presence of reduced copper [26] and ii) the desorption/reduction of strongly bonded oxygen species (’-oxygen) [34]. 

In the H_2_-TPR profile of the BFC3 catalyst, a broad peak between 300 and 500 °C is detected with a well-defined maximum at 320 °C, followed by a shoulder around 380 °C. The first maximum corresponds to the reduction of Cu(II) to Cu(0) and it is better defined than the corresponding to BFC1 due to the higher copper content. As for BFC1, the low H_2_ consumption at T < 300 °C confirms the presence of Fe(IV). The shoulder at 380 °C has to be related with the reduction of Fe(III) to Fe(II) that seems to take place at lower temperature than for BFC1. This fact supports that the formation of metallic copper (which is more easily reduced than iron) promotes the reduction of Fe(III) to Fe(II), as was previously observed for the reduction of manganese species in BaMn_1−x_Cu_x_O_3_ catalysts series [26].

Concerning BFC4, a sharp H_2_ consumption peak with a maximum at 315 °C is found, followed by a low intensity peak with a maximum at around 475 °C. The presence of this well- defined peak, which is ascribed to Cu(II) to Cu(0) reduction, confirms the existence of copper oxide (II) species [34]. In fact, for this catalyst, BaO_x_-CuO_x_ oxide has been detected by XRD and FESEM, thus, the sharp peak corresponds to the reduction of this copper oxide. The second peak has to be due to the Fe(III) reduction to Fe(II) that, for BFC1 and BFC3 catalysts, takes place at lower temperature that for BFC0.

After the analysis of the H_2_-TPR profiles for three catalysts, it can be concluded that the Fe(III) reduction to Fe(II) takes place at similar temperature for BFC1 and BFC4 (450 °C and 475 °C, respectively); this happens at lower a temperature (380 °C) for the BFC3 catalyst, probably due to its higher content of lattice copper (see Table 3).

Concluding, H_2_-TPR results indicate the co-existence of Fe(III) and Fe(IV) and suggest that copper incorporation promotes the reduction of Fe(III) to Fe(II). 

#### 3.1.4. O_2_ Release During Heat-Treatment in He (O_2_-TPD)

In the O_2_ profiles evolved by perovskite mixed oxides during a heat treatment in He (O_2_-TPD), three regions are usually observed [26,39,59,60,61,62,63,64,65,66,67,68]. The lower temperature region, at T < 400 °C corresponds to weakly chemisorbed oxygen upon surface-oxygen vacancies (denoted as oxygen). The intermediate region, between 400 °C and 700 °C, is ascribed to near-surface oxygen associated to lattice defects such as dislocations and grains frontiers (designed as α’ oxygen). Therefore, the presence of α and α‘-oxygen is directly linked with the presence of surface vacancies/defects of oxygen in the structure [64,65,66]. Finally, the oxygen evolved at temperature higher than 700 °C, named β oxygen, is generally related with the lattice oxygen (which comes from the reduction of B cation (Fe in this case) of the perovskite [66]) and it is related with the oxygen mobility and with the inner bulk oxygen vacancies.

**Figure 8:**
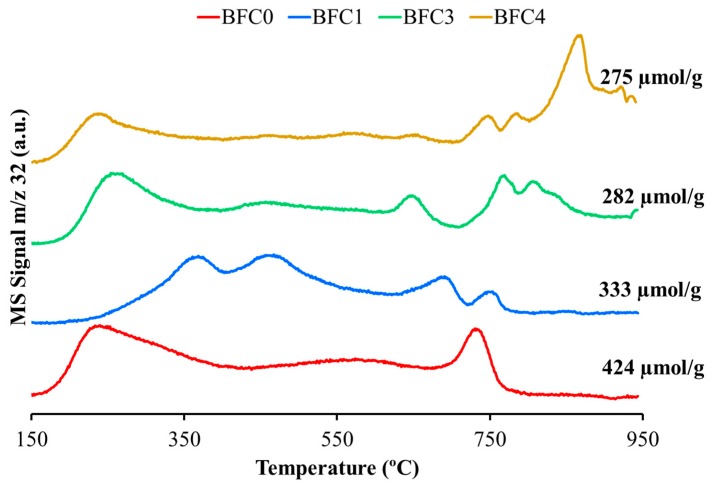
O_2_-TPD profiles for BaFe_1−x_Cu_x_O_3_ catalysts.

Figure 8 shows the O_2_ profiles for the BaFe_1−x_Cu_x_O_3_ catalysts. The O_2_–TPD profiles show that α and α‘-oxygen are mainly evolved by most of the BaFe_1−x_Cu_x_O_3_ catalysts [8,9]. BFC0, BFC3, and BFC4 exhibit a higher oxygen signals than BFC1 and, therefore, higher quantity of surface oxygen vacancies, agreeing with the XPS results (lower O_L_/Cu+Ti+Ba ratio). Regarding α‘-oxygen, BFC1 presents the highest signal, which evidences the great structure distortion (as exhibited by XRD) promoted by a small Cu incorporation. The total quantity of O_2_, calculated from the area under the O_2_ profiles, diminishes as copper content grows: 424 µmol/g cat (BFC0) > 333 µmol/g cat (BFC1) > 282 µmol/g cat (BFC3) > 275 µmol/g cat (BFC4). Thus, as it has been previously published [39], the addition of a dopant seems to stabilize the oxygen bonded to Fe and leads to a decrease in the desorbed O_2_. For BFC4, a grown in the β oxygen has been detected, probably related to the structural modification and the presence of the BaOx-CuOx phase identified by XRD.

The phase composition of BaFe_1−x_Cu_x_O_3_ catalysts after the O_2_-TPD has been determined by XRD (Figure S3 in Supplementary Information). For BFC0 and BFC1 catalysts, the hexagonal perovskite structure is replaced by a monoclinic BaFeO_2.5_ phase (with ordered oxygen vacancies) after losing a fraction of the lattice oxygen. On the contrary, BFC3 and BFC4 catalysts preserve the cubic perovskite structure after O_2_ release. This founding agrees with the conclusions of Huang et al. [39], who pointed out the increase in the structure stability due to the presence of a dopant (copper in our case). Note that the most stable catalysts (BFC3 and BFC4) are those with ideal (cubic) perovskite structure. The higher structural stability in the presence of copper could be relevant for catalytic applications at high temperature.

### 3.2. Catalytic Activity

#### 3.2.1. NO_2_ Generation and Diesel Soot Oxidation

**Figure 9:**
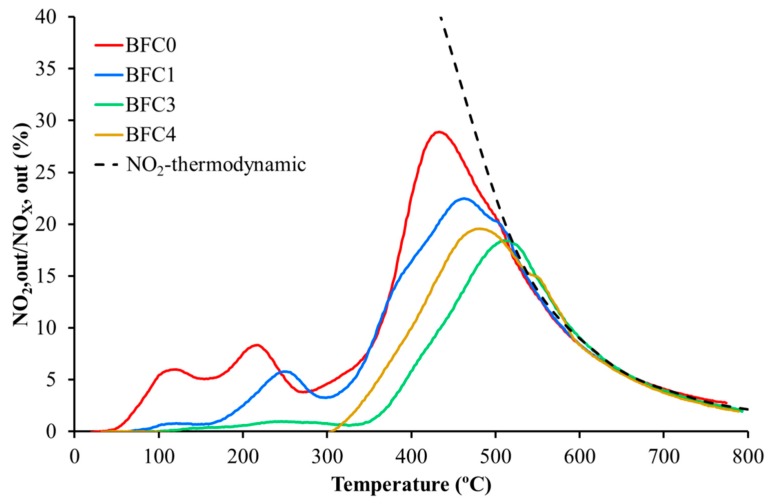
NO_2_ generation profiles in TPR conditions s for BaFe_1−x_Cu_x_O_3_ catalysts.

Figure 9 shows the NO_2_ generation profiles obtained in TPR conditions for BaFe_1−x_Cu_x_O_3_ catalysts including, as reference, the thermodynamic equilibrium profile. As observed for other perovskite-based catalysts [26,34,35,36,37,38,64,67], the thermodynamic equilibrium limits the NO_2_ percentage at T > 500 °C. All catalysts accelerate the NO to NO_2_ oxidation at temperature lower than 500 °C, being the copper-free catalyst (BFC0) the most active. In general terms, the NO_2_ generation follows the same sequence than the amount of oxygen evolved during O_2_-TPD, except for BFC4 catalyst. Note that the two catalysts evolving large amount of and ’-oxygen, that is, BFC0 and BFC1, are also the catalysts generating more NO_2_ at low temperature (T < 300 °C). This is in agreement with the relationship found by Onrubia et al. [64] between the amount of and ’ oxygen evolved by the catalysts (Sr-doped LaBO_3_ (B = Mn or Co perovskites) and the activity for the NO to NO_2_ oxidation. Note that BFC4 shows a slightly higher NO_2_ generation capacity than BFC3, which has to be related with the presence of copper species on the surface (BaOx-CuOx) that also catalyze the NO_2_ production [34].

**Figure 10:**
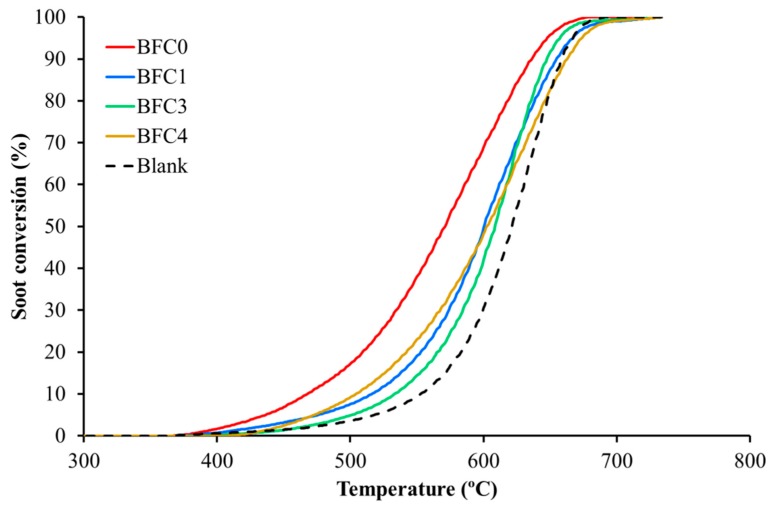
TPR soot conversion profiles in NOx for BaFe_1−x_Cu_x_O_3_ catalysts.

To evaluate the activity of the catalysts for NO_2_-assisted diesel soot oxidation, Temperature Programmed Reactions in a NO/O_2_ atmosphere (see Experimental Section for details) were carried out, and the TPR-NOx soot conversion profiles (calculated based on the amount of CO and CO_2_ evolved) are featured in Figure 10. Relevant data, such as the ignition temperature (T_5%_), the temperature required to reach 50% of soot conversion (T_50%_), and the selectivity to CO_2_, are included in Table 4. It can be concluded that all the catalysts shift the soot conversion profiles to lower temperatures compared to the uncatalyzed reaction (blank corresponding to bare soot) and, consequently, the T_5%_ and the T_50%_ are lower. In agreement with the NO_2_ profiles (Figure 9), BFC0 is the most active catalyst for diesel soot oxidation as the addition of copper decreases the catalyst activity for soot conversion. Moreover, for BFC0 the T_50%_ value is close to 500 °C, thus, this perovskite could be used as potential catalyst for the soot removal from diesel engine exhaust [69]. The decrease in the activity for soot oxidation after the addition of a dopant (copper in our catalysts) was also observed by Huang et al. [39] for Ag-doped LaFeO_3_ catalysts. These authors related the lower activity of Ag-perovskites for soot oxidation with the reduction in the amount of surface oxygen vacancies due to the anchorage of Ag nanoparticles. Furthermore, the reaction rate for methane combustion of a series of oxygen deficient SrFeO_3_ perovskites was related with the quantity of oxygen vacancies in the structure [58]. In fact, a relationship between soot oxidation performance and oxygen vacancies has been published [70]. Thus, in BaFe_1−x_Cu_x_O_3_ catalysts, the decrease in the total amount of O_2_ evolved as copper content increases apparently leads to a decrease in the activity for both NO to NO_2_ and soot oxidation. Note that BFC4 does not match this trend as it shows the lowest T_5%_ y T_50%_ values among the catalysts containing copper (BFC1, BFC3, and BFC4). This catalyst presents the highest fraction of surface copper species, which also catalyzes the NO_2_/soot oxidation reaction [26,34,36], and hence, improves its catalytic performance. Therefore, the activity for NO_2_ generation and the amount of surface copper species seem to determine the catalytic performance. Thus, the highest NO_2_ generation capacity of BFC0 catalyst seems to justify its highest soot oxidation activity, while it is the largest fraction of surface copper species present in BFC4, which seems to justify its higher soot oxidation activity compared to BFC3.

Additionally, the data in Table 4 reveal that, as could be expected [26,34,36,37], all the catalysts show a higher CO_2_ selectivity than the uncatalyzed reaction (bare soot), with BFC4 being the most selective. Thus, CO_2_ selectivity increases with the amount of surface copper species (Table 3) as this metal is a well-known catalyst for CO to CO_2_ oxidation. 

Due to its high activity, the performance of the BFC0 catalyst was deeply analyzed and five consecutives TPR-NOx soot oxidation cycles were carried out using the same portion of catalyst. As the T_50%_ values for the first (543 °C) and fifth cycle (561 °C) are still under those corresponding to the uncatalyzed reaction (612 °C), it can be concluded that the catalyst is not significantly deactivated. In fact, the XRD data (shown in Figure S4 in Supplementary Information) of this used catalyst (after five TPR-NOx cycles) reveal that the hexagonal perovskite structure is not significantly modified. This means that, in the presence of oxygen in the reaction atmosphere, the catalyst keeps its structure and, consequently, its activity for soot oxidation in TPR conditions.

Finally, to complete the BFC0 evaluation, its catalytic performance for NO_2_-assisted diesel soot oxidation in isothermal conditions was determined by carrying out two consecutive soot oxidation experiments at 450 °C. The soot oxidation profiles at 450 °C (featured in Figure S5 in Supplementary Information) show that BFC0 catalyst is able to oxide soot without a significant deactivation and showing a high CO_2_ selectivity (close to 80%). The soot oxidation rate was calculated at the beginning of the reaction, in order to avoid the effect of high soot consumption, as being 1.2 min^−1^, which is not too far from a commercial model Pt/Al_2_O_3_ catalyst (1.8 min^−1^) used in the same experimental conditions. Thus, it seems that BaFeO_3_ perovskite could be a potential catalyst for diesel soot oxidation and, consequently, it could be used as an active phase for DPF.

#### 3.2.2. Soot Oxidation in GDI Conditions

A preliminary study about the use of BaFe_1−x_Cu_x_O_3_ perovskites to catalyze the oxidation reaction of soot under the highest demanding GDI exhaust requirements (regular stoichiometric GDI operation, i.e., 0% O_2_) revealed that these oxides could be used as active phase for GPF [35]. It was concluded that the copper content has an essential role on the performance of the BaFe_1−x_Cu_x_O_3_ catalysts for soot oxidation, agreeing with previous reports focused on diesel soot removal [10,26,34,36,37,38] and with the report for copper-supported ceria-zirconia catalysts for soot oxidation in GDI conditions [10]. These results confirm that the higher soot conversion presented by BFC4 with respect to the catalysts with lower copper content (BFC1 and BFC3) is linked both to the largest amount of β oxygen evolved by this catalyst, and with the presence of surface copper species (as BaO_x_-CuO_x_) [10,34,36,37,38]. 

**Figure 11:**
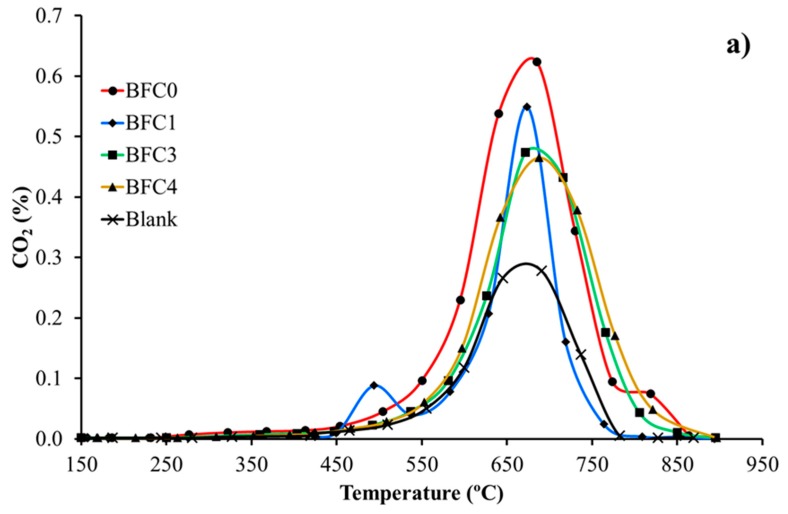

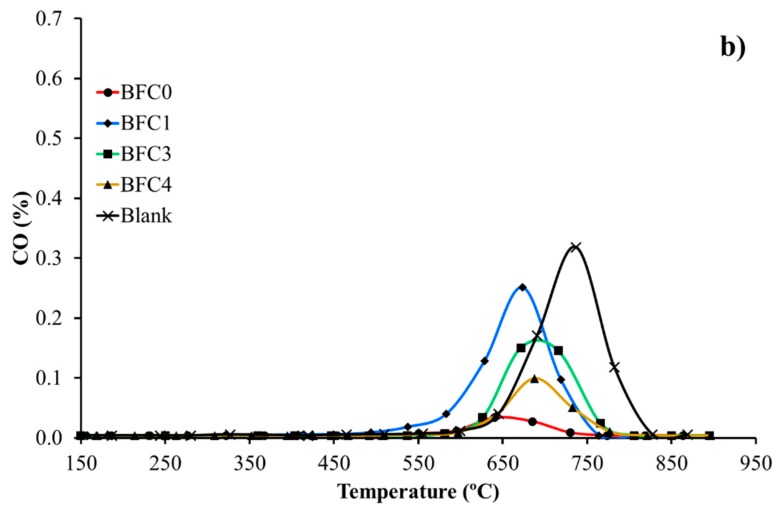
CO_2_ (**a**) and CO (**b**) profiles during TPR soot oxidation in 1% O_2_ for BaFe_1−x_Cu_x_O_3_ catalysts.

To further analyze the performance of these BaFe_1−x_Cu_x_O_3_ perovskites in GDI exhaust conditions, a study using 1% O_2_ in He (which represents the named “fuel cuts” GDI exhaust conditions) has been developed [9]. Figure 11 shows the profiles of CO_2_ and CO evolved during temperature programmed reaction experiments, including the profiles for the uncatalyzed reaction (blank corresponding to bare soot), as reference. Note that in the presence of BaFe_1−x_Cu_x_O_3_ catalysts, the amount of CO decreases and the amount of CO_2_ increases. This means that, as could be expected [26,34,36,37], and as was observed during soot conversion reaction in NOx atmosphere, the catalysts increase the selectivity to CO_2_ from 56% for bare soot to 93% for BFC0, 70% for BFC1, 79% for BFC3, and 87% for BFC4. As for NO_2_ assisted diesel soot oxidation, the BFC0 catalyst is the most active and the addition of copper seems to decrease the ability of the catalysts to improve the CO_2_ selectivity. Additionally, as it has been deduced from soot conversion results in NOx atmosphere (see Table 4) that BFC4 presents the highest CO_2_ selectivity among the copper containing catalysts due to the presence of the surface copper species.

**Figure 12:**
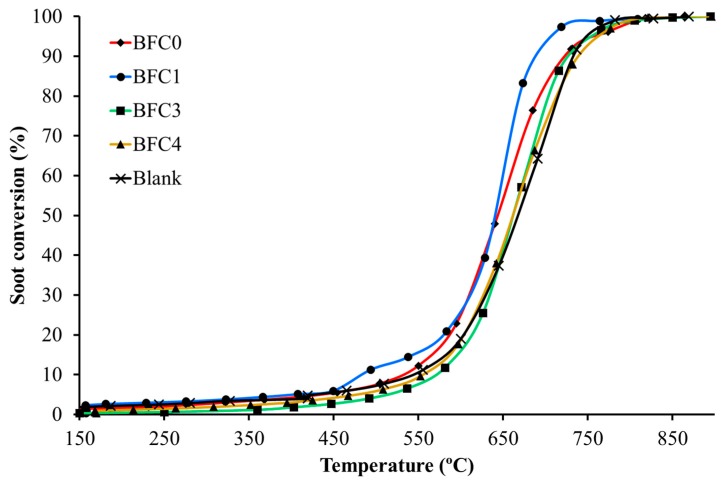
TPR soot conversion profiles in 1% O_2_ for BaFe_1−x_Cu_x_O_3_ catalysts.

Figure 12 shows the soot conversion profiles in 1% O_2_, calculated based on the amount of evolved CO and CO_2_ featured in Figure 11.

In general terms, and in agreement with previous results for BaMn_1−x_Cu_x_O_3_ catalysts [26], the catalytic effect is less relevant than the observed for NO_2_-assisted diesel soot oxidation. Thus, only BFC0 and BFC1 catalyze the soot oxidation with oxygen as the conversion profiles are shifted to lower temperature with respect to bare soot for these two catalysts. As observed for NO_2_ generation (see previous section), and in agreement with published conclusions [39,58,70], the soot conversion with oxygen follows the same trend than the amount of oxygen evolved during O_2_-TPD (Figure 8) as the most active catalysts (BFC0 and BFC1) are those generating the largest amount of oxygen. In fact, the highest activity of BFC1 at low temperature, i.e., at T < 600 °C approximately, seems to be related with the largest amount of and ’ evolved (Figure 8). Thus, it seems that a similar reaction pathway is followed by soot oxidation with oxygen and with NO_2_-assisted diesel soot oxidation, even though the catalytic effect is more relevant for the latter reaction than for the former. Hence, it could be concluded that the BaFe_1−x_Cu_x_O_3_ perovskites catalyze more effectively the NO_2_-soot reaction than the O_2_-soot reaction. Additionally, the comparison of these results with the obtained in the most demanding GDI conditions (0% O_2_) [35] reveals that copper has an essential role on the performance of the BaFe_1−x_Cu_x_O_3_ catalysts for soot oxidation only in the absence of oxygen in the reaction atmosphere. 

Summarizing, the activity data above discussed reveals that the BaFe_1−x_Cu_x_O_3_ perovskites catalyze both, the NO_2_-assisted diesel soot oxidation (500 ppm NO, 5% O_2_) and, to a lesser extent, the soot oxidation in the “fuel cut” GDI exhaust conditions (1% O_2_). BFC0 is the most active catalyst as the activity seems to be mainly related with the amount of O_2_ evolved during an O_2_-TPD, which decreases with copper content. 

## 4. Conclusions

The results obtained for the BaFe_1−x_Cu_x_O_3_ (*x* = 0, 0.1, 0.3 and 0.4) catalyst series allows us to conclude that:

- Partial substitution of iron by copper in the lattice of a BaFeO_3_ perovskite generates a distortion of the hexagonal perovskite structure for the lowest copper content catalyst (BFC1), and a change to cubic structure for the catalysts with higher copper content (BFC3 and BFC4).
- The amount of copper inserted into the perovskite framework achieve a maximum for the highest copper content catalyst (BFC4), which provokes the presence of BaOx-CuOx as a minority segregated phase.
- The positive charge deficiency due to the partial substitution of Fe by Cu seems to be balanced by the oxidation of Fe(III) to Fe(IV) in the BFC1 perovskite and by the generation of additional oxygen vacancies/defects, for BFC3 and BFC4 catalysts.
- BaFe_1_-xCuxO_3_ perovskite catalyze both the NO_2_-assisted diesel soot oxidation (500 ppm NO, 5% O_2_) and, to a lesser extent, the soot oxidation in the high demanding GDI exhaust conditions (1% O_2_)
- BFC0 is the most active catalyst for both oxidation reactions. The activity seems to be mainly related with the amount of O_2_ evolved during an O_2_-TPD, which decreases with the copper content of the catalyst.

## Figures and Tables

**Table 1:** Molecular composition, specific surface area, copper content, and Goldschmidt tolerance factor (*t)* * values for BaFe_1−x_Cu_x_O_3_ catalysts.

Catalyst	Molecular Composition	BET Specific Surface Area (m^2^/g) *	Nominal Cu (wt %)	ICP Cu (wt %)	*T* (Fe^3+^) **	*t* (Fe^4+^) **
BFC0	BaFeO_3_	4	--	--	1.09	1.12
BFC1	BaFe_0.9_Cu_0.1_O_3_	1	2.5	2.5	1.09	1.12
BFC3	BaFe_0.7_Cu_0.3_O_3_	1	7.7	7.0	1.08	1.10
BFC4	BaFe_0.6_Cu_0.4_O_3_	3	9.1	9.1	1.07	1.09

* In the range of experimental detection limit. ** Calculated as: $t=\frac{R_{\mathrm{Ba}}+R_{O}}{\sqrt{2}\cdot \left((\left(1-x)\cdot R_{\mathrm{Fe}}+{\mathrm{xR}}_{\mathrm{Cu}}\right)+R_{O}\right)}$.

**Table 2:** XRD characterization data of BaFe_1−x_Cu_x_O_3_ catalysts.

Catalyst	XRD Phase Identification	Average Crystal Size (nm) *	*a* (Å) *	*c* (Å) *
BFC0	BaFeO_2.67_, hexagonal	17.0	5.684	13.925
BFC1	BaFeO_2.67_, hexagonal	12.4	5.667	13.908
BFC3	BaFeO_3,_ cubic	14.3	4.019	-
BFC4	BaFeO_3,_ cubic, Ba_0.9_Cu_1.06_O_2.43_	16.9	4.018	-

* Calculated using the main XRD perovskite peak.

**Table 3:** XPS characterization data of BaFe_1−x_Cu_x_O_3_ catalysts.

Catalyst	Cu/ Ba+Fe+Cu (nominal)	Cu/ Ba+Fe+Cu (XPS)	O_L_/ Ba+Fe+Cu (XPS)
FC0	-	-	1.30
BFC1	0.05	0.03	1.70
BFC3	0.15	0.09	1.10
BFC4	0.20	0.21	1.10

**Table 4:** Data for NO_2_-assisted diesel soot oxidation in TPR conditions BaFe_1−x_Cu_x_O_3_ catalysts.

Catalysts	T_5%_ (°C)	T_50%_ (°C)	CO_2_ Selectivity (%)
Bare soot	480	612	41
BFC0	430	543	51
BFC1	455	605	70
BFC3	480	605	66
BFC4	454	590	90

## Supplementary Materials

The following are available online at https://www.mdpi.com/2079-4991/9/11/1551/s1, Figure S1: FESEM pictures for BaFe1-xCuxO3; Table S1: EDX data (atomic percentage) for BFC0 and BFC4 catalysts; Figure S2: Wagner (chemical state) plot for BaFe_1−x_CuxO_3_ catalysts; Figure S3: DRX patterns after O_2_-TPD for BaFe_1−x_CuxO_3_ catalysts (dotted lines). As reference, XRD patterns of fresh catalysts (solid line) have been included; Figure S4: DRX patterns after TPR-NOx with soot for BFC0 catalyst (dotted line). As reference, XRD pattern of fresh catalyst (solid line) has been included; Figure S5: Soot conversion profiles at 450 °C for BFC0.
