# Supplementary material for: BaFe1−xCuxO3 Perovskites as Active Phase for Diesel (DPF) and Gasoline Particle Filters (GPF)
Source: Nanomaterials (Basel). 2019 Oct 31;9(11):1551. doi: 10.3390/nano9111551 (PMC6915380; doi:10.3390/nano9111551)
Supplement: Supplementary file 1 [file nanomaterials-09-01551-s001.pdf]

Supplementary Materials

$BaFe_{1-x}Cu_xO_3$ Perovskites as Active Phase for Diesel (DPF) and Gasoline Particle Filters (GPF)

Verónica Torregrosa-Rivero, Carla Moreno-Marcos, Vicente Albaladejo-Fuentes, María-Salvadora Sánchez-Adsuar and María-José Illán-Gómez *

Carbon Materials and Environment Research Group, Department of Inorganic Chemistry, Faculty of Science, University of Alicante, Av. Alicante s/n, San Vicente del Raspeig, Alicante 03690, Spain; vero.torregrosa@ua.es (V.T.-R.); carlamorenomarcos1@gmail.com (C.M.-M.); vicentealbaladejo@gmail.com (V.A.-F.); dori@ua.es (M.-S.S.-A.);

* Correspondence: illan@ua.es; Tel.: +34-965-903-975

a) BFC0

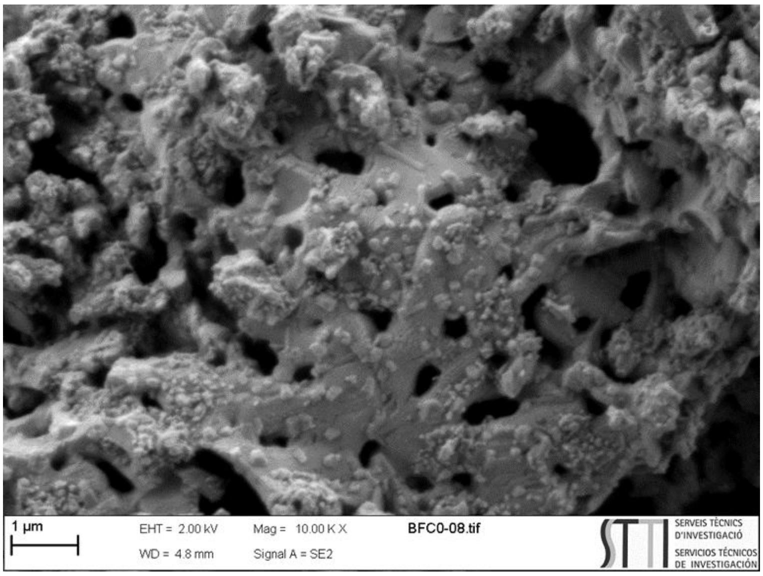

b) BFC1

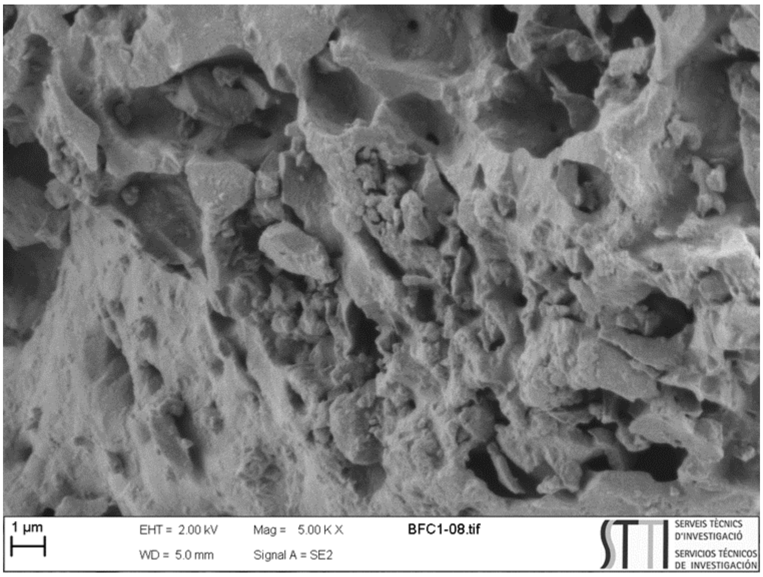

c) BFC3

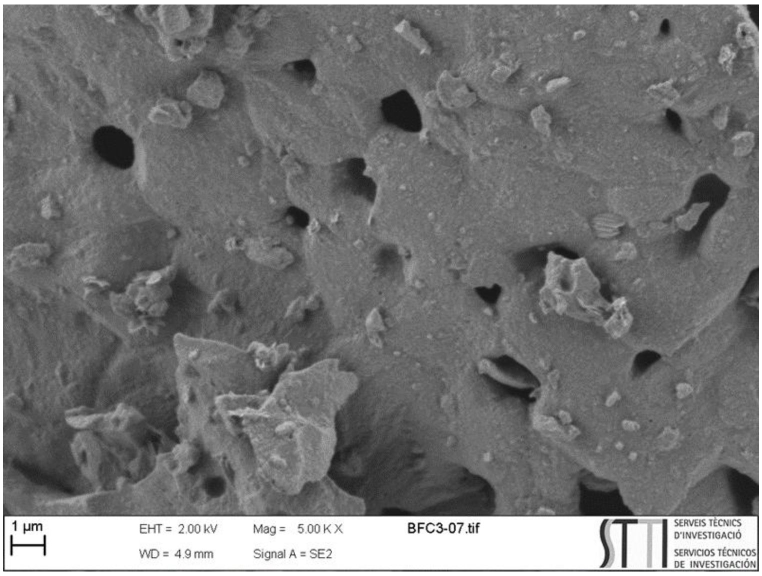

d) BFC4

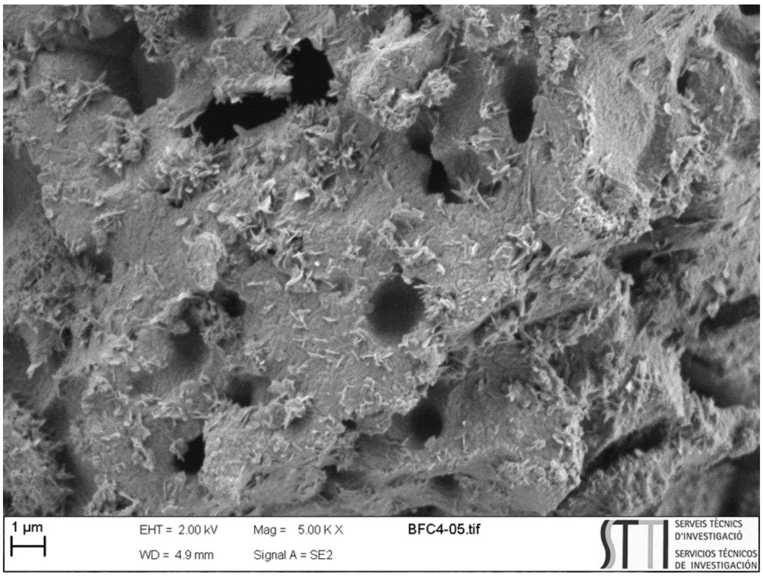

Figure S1. FESEM pictures for $BaFe_{1-x}Cu_xO_3$.

Table S1. EDX data (atomic percentage) for BFC0 and BFC4 catalysts.

Catalyst	% O	% Ba	% Fe	% Cu
BFC0	42.3	6.3	6.3	---
BFC4	46	9.5	6.3	4.3

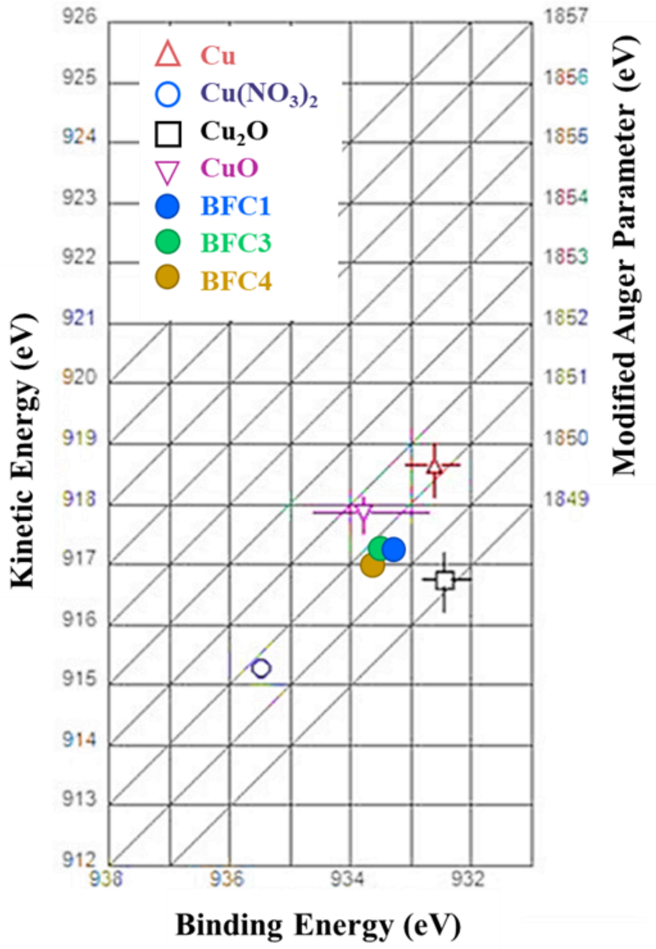

Figure S2. Wagner (chemical state) plot for $BaFe_{1-x}Cu_xO_3$ catalysts.

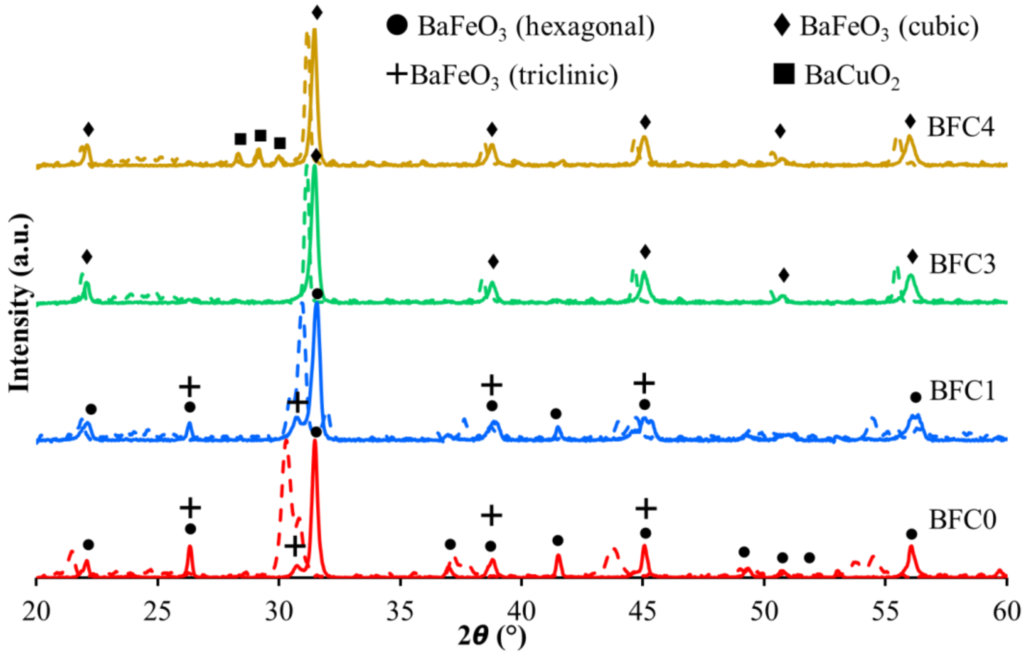

Figure S3. DRX patterns after O_2-TPD for $BaFe_{1-x}Cu_xO_3$ catalysts (dotted lines). As reference, XRD patterns of fresh catalysts (solid line) have been included.

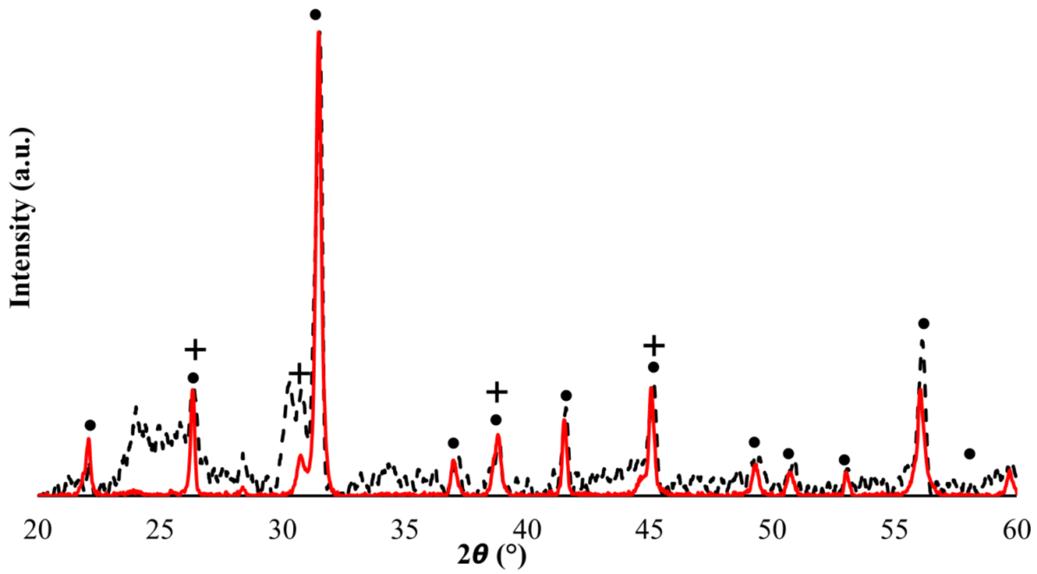

Figure S4. DRX patterns after TPR-NOx with soot for BFC0 catalyst (dotted line). As reference, XRD pattern of fresh catalyst (solid line) has been included.

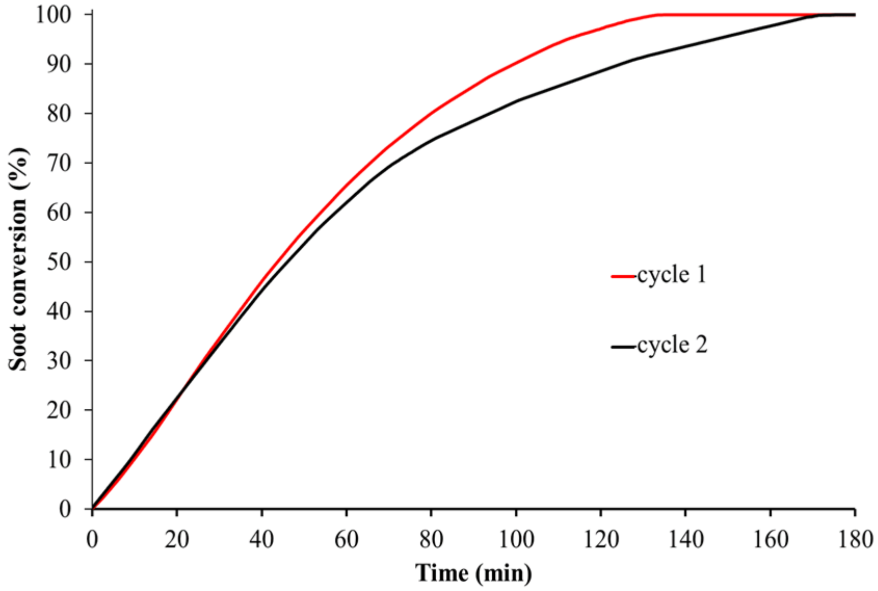

Figure S5. Soot conversion profiles at 450 °C for BFC0
